# Supplementary material for: Application of machine learning and complex network measures to an EEG dataset from ayahuasca experiments
Source: PLoS One. 2022 Dec 16;17(12):e0277257. doi: 10.1371/journal.pone.0277257 (PMC9757568; doi:10.1371/journal.pone.0277257)
Supplement: S1 Appendix — (PDF) [file pone.0277257.s001.pdf]

A EEG database electrodes

Figure 13 showing the head model schematic with electrodes from the EEG database used in

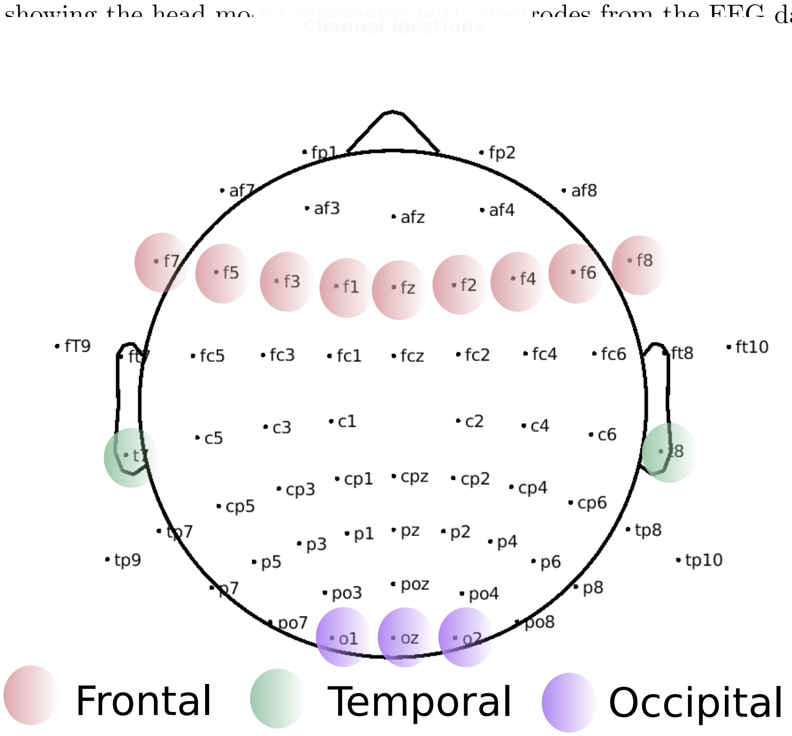

Fig 13. Head model for all electrodes used in the present work. The brain regions modulated after using ayahuasca, according to literature, are the frontal, temporal, and occipital lobes, highlighted in the Fig in pink, green and purple, respectively. Developed by the authors using MNE-Python [1].

B Hyperparameter values of Grid Search classifier

We used grid search as an optimization method to achieve good performance, which is a method that exhaustively tests all possible combinations of values for each hyperparameter considered—the Table 3 containing each classifier, the hyperparameters, and the values used in the grid search tunning.

Table 3. Table containing the hyperparameters for each classifier using the Grid search optimizer.

Classifier	Hyperparameters and description	Values
**RF**	- max_depth: The maximum depth of the tree.	[1,2,5,10,20,80]
	- max_features: The number of features to consider when looking for the best split.	[2, 3,5,10]
	- min_samples_leaf : The minimum number of samples required to be at a leaf node.	[1,2,3, 4, 5]
	- min_samples_split: The minimum number of samples required to split an internal node.	[1,2,8, 10, 12,20]
	- n_estimators: The number of trees in the forest.	[1,2,3,5,10, 30,50,100, 200, 300,500]
**SVM**	-kernel: Specifies the kernel type to be used in the algorithm.	[rbf, linear]
	-gamma: Kernel coefficient.	[1e-3, 1e-4]
	-C: Regularization parameter.	[1, 10, 100, 1000]
**NB**	-var_smoothin:Portion of the largest variance of all features that is added to variances for calculation stability.	range 1e-09 to 1
**MLP**	- activation: Activation function for the hidden layer.	[identity, logistic, tanh, relu]
	- solver: The solver for weight optimization.	[lbfgs, sgd, adam]
	- alpha: L2 penalty (regularization term) parameter.	[0.0001,1e-5,0.01,0.001]
	- batch_size: Size of minibatches for stochastic optimizers.	[1000,5000]
	- learning_rate: Learning rate schedule for weight updates.	[constant, invscaling, adaptive]
	- learning_rate_init: The initial learning rate used.	[0.001,0.01,0.1,0.2,0.3]
**SGD**	- loss: The loss function to be used	[hinge, log, squared_hinge, modified_huber]
	- alpha: Constant that multiplies the regularization term. The higher the value, the stronger the regularization.	[0.0001, 0.001, 0.01, 0.1]
	- penalty: The penalty (regularization term) to be used.	[l2, l1, none]
**LR**	- C: Each of the values in Cs describes the inverse of r egularization strength.	range 0.001 to 1000
	- penalty: Specify the norm of the penalty.	[l1, l2]
**XGBoost**	- learning_rate: Learning rate shrinks the contribution of each tree.	[0.01, 0.025, 0.05, 0.075, 0.1, 0.15, 0.2]
	- min_samples_split: The minimum number of samples required to split an internal node.	range 0.1 to 0.5
	- min_samples_leaf: The minimum number of samples required to be at a leaf node.	range 0.1 to 0.5
	- max_depth: The maximum depth of the individual r egression estimators.	[3,5,8]
	- max_features: The number of features to consider when looking for the best split.	[log2, sqrt]
	- criterion: The function to measure the quality of a split.	[friedman_mse, mae],
	- subsample: The fraction of samples to be used for fitting the individual base learners.	[0.5, 0.618, 0.8, 0.85, 0.9, 0.95, 1.0],
	-n_estimators: The number of boosting stages to perform.	[10, 100. 1000, 10000]

C Results of all classifier in the complex network measures

In the present study, we tested several machine learning algorithms whose performance is shown in the Table 4.

Table 4. Performance of all classifiers applied to the network measurements. In bold the best performance referring to the SVM classifier.

Classifier	Subset	AUC	Acc.	F1 score	Recall	Precision
**RF**	**Train**	0.69	0.75	0.70	0.69	0.72
	**Test**	0.44	0.58	0.37	0.44	0.32
**SVM**	**Train**	**0.79**	**0.81**	**0.78**	**0.79**	**0.79**
	**Test**	**0.75**	**0.83**	**0.79**	**0.75**	**0.90**
**NB**	**Train**	0.83	0.86	0.84	0.83	0.85
	**Test**	0.50	0.67	0.40	0.50	0.33
**MLP**	**Train**	1.00	1.00	1.00	1.00	1.00
	**Test**	0.50	0.67	0.40	0.50	0.33
**SGD**	**Train**	0.75	0.78	0.75	0.75	0.75
	**Test**	0.56	0.67	0.55	0.56	0.60
**LR**	**Train**	0.83	0.86	0.84	0.83	0.85
	**Test**	0.63	0.75	0.62	0.63	0.86
**XGBoost**	**Train**	0.98	0.97	0.97	0.98	0.96
	**Test**	0.38	0.50	0.33	0.38	0.3

D Deep learning results

Additionally, Pearson's connectivity matrix was used as an input to the deep learning algorithm implemented in [1] with random research tuning. The results are shown in Table 5 and Fig 14, in which it can be seen that it was possible to capture the alteration due to ayahuasca without an indication of underfitting and overfitting. Also, the python code used for the analysis is available at:
`https://github.com/Carol180619/Paper-ayahuasca.git`.

Table 5. Results were obtained by the use of the deep learning model.

Subset	AUC	Acc.	F1 score	Recall	Precision
**Train**	1.00	1.00	1.00	1.00	1.00
**Test**	0.96	0.94	0.94	0.94	0.94

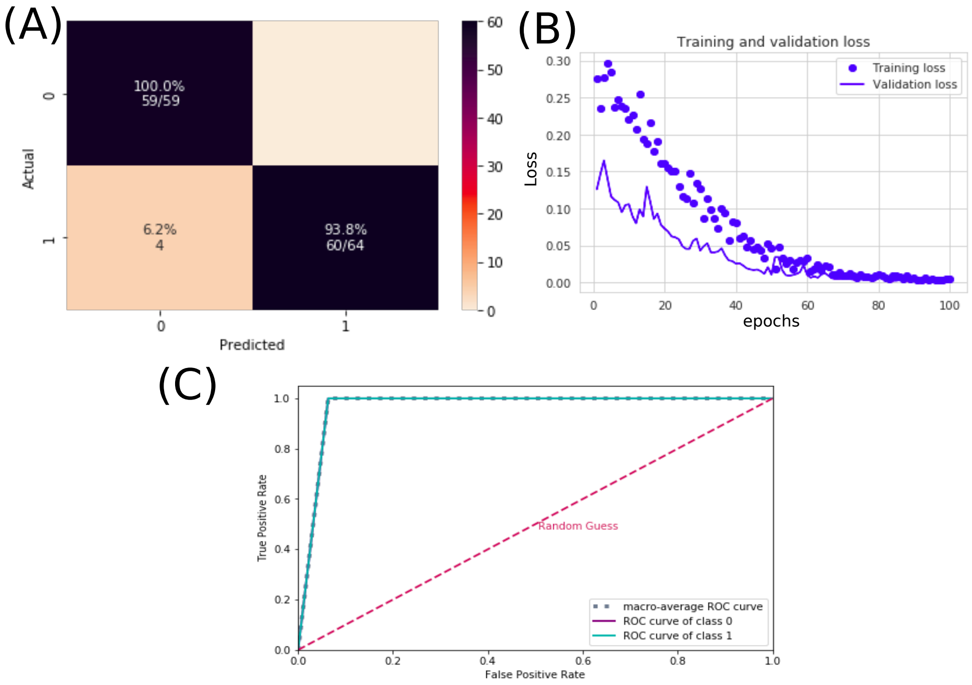

Fig 14. Results were obtained by the use of the deep learning model. A) The confusion matrix indicates a true negative rate of 93.8% (blue according to the color bar) and a true positive rate of 100.0% (blue according to the color bar). B) Loss value over the training data (blue dots points) and validation data (blue line) after each epoch; in this curve, the error loss decreases, indicating no underfitting or overfitting. C) ROC curve of class 0 (without ayahuasca) and class 1 (with ayahuasca). The gray dotted curve is the macro-average accuracy (area under curve = 0.96), and the pink one is the random classifier.

Reference

1. Gramfort A, Luessi M, Larson E, Engemann DA, Strohmeier D, Brodbeck C, et al. MEG and EEG data analysis with MNE-Python. Frontiers in neuroscience. 2013;7:267.
