# Supplementary material for: Application of machine learning and complex network measures to an EEG dataset from ayahuasca experiments
Source: PLoS One. 2022 Dec 16;17(12):e0277257. doi: 10.1371/journal.pone.0277257 (PMC9757568; doi:10.1371/journal.pone.0277257)
Supplement: Attachment — Submitted filename: Author_response_to_review_comments_ayahuasca_paper_FINAL.pdf [file pone.0277257.s002.pdf]

Authors' Response to Reviews of

Application of machine learning and complex network measures to an EEG dataset from ayahuasca experiments

Caroline L. Alves, Rubens Gisbert Cury, Kirstin Roster, Aruane M. Pineda, Francisco A. Rodrigues, Christiane Thielemann, Manuel Ciba
PLOS ONE,

RC: ***Reviewers' Comment***, AR: Authors' Response, ☐ Manuscript Text

We would like to thank the reviewer for their careful consideration of our manuscript and recommendation for acceptance after major revision. The comments have been very useful and have helped us improve the analysis and clarity of explanation in the manuscript. We provide point-by-point responses to each comment below.

1. Reviewer 1

RC: ***My major concern, because of which I answered the question about technical soundness with "partially" is that in order to assist reproduction and independent validation of the results, and to promote the use of the same methodology in other studies, I think the Authors should publish their implementation of the methods, including preprocessing steps (i.e., source codes of the software they implemented during their research work).***

AR: Thank you for your feedback. We have saved all of the code used in a private GitHub account (including the new codes referring to the appendix section with our deep learning code) and will make it public once the article is published. The python code used for the analysis will be available at `https://github.com/Carol180619/Paper-ayahuasca.git`.

RC: ***As for the language, the Authors should keep in mind that PLOS ONE does not copyedit manuscripts, therefore they should proofread their work carefully. For example, special attention should be payed to references, both references to subsections (that are often broken in the current version of the manuscript) and references to the literature. See for example: "...known from schenberg2015acute" (third line in Section 3.1.1).***

AR: Thanks for pointing out the grammatical errors. We have grammatically revised the entire text with the assistance of language professionals.

RC: ***As for the methodology, while I understand that the Authors used standard machine learning methods, both for classification of the data, as well as for the other subtasks, such as community detection, it would be worth to discuss recent approaches that were designed to deal with brain activity data. See, for example:***

`http://biointelligence.hu/pdf/saci2020_5_buza.pdf`

`https://link.springer.com/chapter/10.1007/978-3-319-45823-6_59`

AR: Thank you very much for the punctuation. The methodology section has been improved. Thank you also for sharing this reference. We have included it in the manuscript in the Introduction section.

2. Reviewer 2

RC: ***The current version is not at the journal level.***

AR: Thanks for this observation. We have grammatically revised the entire text with the assistance of language professionals.

RC: ***Difficult to understand.***

AR: Thanks for this observation. We revised the entire text with the help of language specialists, and we believe it is now intelligible.

RC: ***In the related work section, the authors have added short comments to new references; among these, more details of the method proposed in the above-cited paper have to be reported. Should be better explained to outline the limitations of the approaches.***

AR: Thank you very much for your comment. We have rewritten the Introduction and Methodology section, including more information about the methods used.

RC: ***In the Literature review section, the authors should find out the difference between the proposed model and the existing methods should be further described.***

AR: Thank you very much for your observation. We have rewritten the Introduction and included the following paragraph:

"The purpose of this study is to determine whether it is possible to automatically detect the changes in brain activity after intake of ayahuasca with machine learning methods using the following data abstraction levels for the input: (A) raw EEG time series, (B) the correlation between the EEG electrodes as used in (A) represented by a connectivity matrix, and (C) complex network measures extracted from (B). In contrast to articles in the literature that use only one of these levels of abstraction, this study uses all three levels. In addition, we define which of these abstraction levels is most appropriate for capturing ayahuasca-induced brain changes. The SHAP value method has also been shown to be more effective than the studies cited above in identifying the best brain regions, the best connections between the brain regions, and the best measures of complex networks, which can be used to assess the effects of the psychedelic substance on the brain. A final result of this research was the creation of new measures that have never been used before within the literature, which can be used as input to machine learning algorithms in order to assess the size of community structures. "

RC: ***The paper's presentation should be improved. It is difficult to understand.***

AR: Thank you for this observation. We proofread the article text in its entirety and rewrote the abstract, introduction, methodology, and conclusion in more detail. We believe the article is now more intelligible.

RC: ***Your abstract does not highlight the specifics of your research or findings.***

AR: Thank you for this observation. We rewrote the abstract to emphasize the important findings.

RC: ***The introduction should be rewritten to clarify its message. Drawbacks of former proposals should be clearly indicated and innovations and new ideas highlighted.***

AR: Thank you very much for your observation. We have rewritten the Introduction and included the following paragraph:

"The purpose of this study is to determine whether it is possible to automatically detect the changes in brain activity after intake of ayahuasca with machine learning methods using the following data abstraction levels for the input: (A) raw EEG time series, (B) the correlation between the EEG electrodes as used in (A) represented by a connectivity matrix, and (C) complex network measures extracted from (B). In contrast to articles in the literature that use only one of these levels of abstraction, this study uses all three levels. In addition, we define which of these abstraction levels is most appropriate for capturing ayahuasca-induced brain changes. The SHAP value method has also been shown to be more effective than the studies cited above in identifying the best brain regions, the best connections between the brain regions, and the best measures of complex networks, which can be used to assess the effects of the psychedelic substance on the brain. A final result of this research was the creation of new measures that have never been used before within the literature, which can be used as input to machine learning algorithms in order to assess the size of community structures. "

RC: ***Problem statement and objective are not clear. The authors should give a more accurate description of the existing methods.***

AR: Thank you for the observation. We have rewritten the current work's objectives and we believe that it is now more understandable.

RC: ***Tables figures present a lot of statistics but need a more detailed explanation (which is missing in the text).***

AR: Thank you for the observation. We made improvements and we believe it is now more clearly described.

RC: ***I feel that more explanation would be needed on how the proposed method is performed.***

AR: Thank you for the observation. The methodology section has been improved with more details. Also, we have saved all of the code used in a private GitHub account (including the new codes referring to the appendix section with our deep learning code) and will make it public once the article is published.

RC: ***If no one has proposed a method like the proposed algorithm, this claim should be highlighted much more. Else, it should be indicated who has done this, and it should be indicated what the innovations of the current paper are.***

AR: Thank you very much for your observation. We have rewritten the Introduction and included the following paragraph:

"The purpose of this study is to determine whether it is possible to automatically detect the changes in brain activity after intake of ayahuasca with machine learning methods using the following data abstraction levels for the input: (A) raw EEG time series, (B) the correlation between the EEG electrodes as used in (A) represented by a connectivity matrix, and (C) complex network measures extracted from (B). In contrast to articles in the literature that use only one of these levels of abstraction, this study uses all three levels. In addition, we define which of these abstraction levels is most appropriate for capturing ayahuasca-induced brain changes. The SHAP value method has also been shown to be more effective than the studies cited above in identifying the best brain regions, the best connections between the brain regions, and the best measures of complex networks, which can be used to assess the effects of the psychedelic substance on the brain. A final result of this research was the creation of new measures that have never been used before within the literature, which can be used as input to machine learning algorithms in order to assess the size of community structures. "

RC: ***Improve text formatting.***

AR: Thank you for this observation. The text formatting have been improved.

RC: ***Why have not been used other DL based methods?***

AR: Thank you for this observation. We also implemented a more robust deep learning algorithm with positive results (see appendix C). We have saved all of the code used in a private GitHub account (including the new codes referring to the appendix section with our deep learning code) and will make it public once the article is published. The python code used for the analysis will be available at `https://github.com/Carol180619/Paper-ayahuasca.git`.

RC: ***Please, also provide a paragraph with three to five clear positive impacts of your method.***

AR: Thank you for this observation. We have rewritten the Introduction and included the following paragraph:

"The purpose of this study is to determine whether it is possible to automatically detect the changes in brain activity after intake of ayahuasca with machine learning methods using the following data abstraction levels for the input: (A) raw EEG time series, (B) the correlation between the EEG electrodes as used in (A) represented by a connectivity matrix, and (C) complex network measures extracted from (B). In contrast to articles in the literature that use only one of these levels of abstraction, this study uses all three levels. In addition, we define which of these abstraction levels is most appropriate for capturing ayahuasca-induced brain changes. The SHAP value method has also been shown to be more effective than the studies cited above in identifying the best brain regions, the best connections between the brain regions, and the best measures of complex networks, which can be used to assess the effects of the psychedelic substance on the brain. A final result of this research was the creation of new measures that have never been used before within the literature, which can be used as input to machine learning algorithms in order to assess the size of community structures. "

RC: ***The authors should further detail the preparation of the dataset.***

AR: Thank you for this observation. We have provided more information about the database utilized, as well as the matrices generated in the present work are now in the public repository. Pearson's connection matrix of the EEG experiments of subjects who ingested Ayahuasca at the time after the psychedelic activation time can be found in: Alves, Caroline (2022): With-ayahuasca. figshare. Dataset. `https://doi.org/10.6084/m9.figshare.21082513.v1`. Pearson's connection matrix of the EEG experiments of subjects who ingested Ayahuasca at the time before the psychedelic activation time: Alves, Caroline (2022): No-ayahuasca. figshare. Dataset. `https://doi.org/10.6084/m9.figshare.21082531.v1`.

RC: ***The figures must be in better format resolution.***

AR: Thank you for this observation. We have modified some images to make them more understandable. Also, all images have been exported in a higher resolution.

RC: ***How to set the parameters of DL algorithms.***

AR: Thank you for this observation. The grid search for the ML algorithms was used as hyperparameter tuning (whose hyperparameters are described in Appendix B). However, for better clarity, we have saved all of the code used in a private GitHub account (including the new codes referring to the appendix section with our deep learning code) and will make it public once the article is published. In addition, the python code used for the analysis will be available at `https://github.com/Carol180619/Paper-ayahuasca.git`.

RC: ***There are no real insightful conclusions drawn from the study and no suggestions for practical use of the results. Therefore, the conclusion section should be totally rewritten in order to:***

- ***You must more clearly highlight the theoretical and practical implications of your research.***

- *Discuss research contributions, Indicate practical advantages, and discuss research limitations.*
- *supply solid and insightful future research suggestions, and references must be updated.*

AR: Thank you for this observation, we rewrite the conclusion to address the points raised. We believe the conclusion is now more understandable and comprehensive.

3. Reviewer 3

The authors studied brain activity changes under ayahuasca by using machine learning and complex network features and showed the high performance of SVM to predict the brain activity change. The research topic is interesting, and the manuscript is written well. However, I have major concerns about the method and results.

3.1. Major comments

RC: *Is this the first study to investigate brain activity under ayahuasca? If not so, the authors should introduce them in Introduction section. Even if so, other previous studies on relationship between brain characteristics (e.g., brain activity, functional connectivity) and medical herbs should be cited in Introduction. Moreover, the authors should describe what kind of results would be expected based on the previous studies.*

AR: Thank you for the suggestion. We have inserted the following paragraph in the Introduction: "In addition, the data used here are from [1], in which EEG data from subjects who ingested ayahuasca were analyzed. This study observed slow-gamma power increases at the left Centro-parietal-occipital, left frontotemporal, and right frontal cortices. In contrast, fast-gamma increases were significant at the left Centro-parieto-occipital, left frontotemporal, right frontal, and right parieto-occipital cortices due to ayahuasca ingestion. As a result, this study concentrated solely on the changes in frequency bands caused by the use of the psychedelic substance."

[1] Schenberg, Eduardo Ekman, et al. "Acute biphasic effects of ayahuasca." PloS one 10.9 (2015): e0137202.

RC: *Why did some ML methods fail to predict test data? For example, XGBoost had remarkably low performance for test dataset.*

AR: Thank you very much for your concern. We believe that SVM performed better than the other algorithms because the problem at hand was high dimensional compared to the number of instances analyzed, as there were 62 electrodes as features. Moreover, it is known that SVM is effective in cases where the number of dimensions is larger than the number of samples. However, we also implemented a more robust deep learning algorithm with positive results (see appendix C).

RC: *The authors did not analyze control experiment data. I'm wondering if the result can be obtained even in without-drug conditions. For example, the authors cannot exclude a possibility that the prediction was based on the characteristics along time (e.g., change due to tiredness).*

AR: Thank you very much for your concern. We believe that the results were due to the use of ayahuasca since, in the original article, it was described that all participants reported noticeable changes in their normal state of consciousness after ingesting ayahuasca. Therefore, we have added this observation in subsection

3.1, where the database is described.

3.2. Minor comments

RC: ***3.2.1: LG -> LR***

AR: ***Thank you very much for this observation, we have changed the abbreviation LG to LR.***

RC: ***Abstract: remove new lines***

AR: ***Thank you very much for this observation, we have removed this lines.***

4. Reviewer 4

Thank you for providing opportunity to review this manuscript. Outcome of the study is interesting. The general impression of this manuscript should follow proper order. Such as:

RC: ***In introduction(page2), it would be nice the research questions are in sentences not in bullet form.***

AR: ***Thank you for this observation. We removed the bullets and described our research questions in paragraph text.***

RC: ***Data (page2) should move to the Material and Methods (in page3).***

AR: ***Thank you for this observation. We have relocated the data section under Material and Methods section.***

RC: ***The biggest concern is that methodology of the data collection utilized. 16 healthy males and females patients were selected for the study. How were these healthy patients selected? 32 patients were appropriate number? What criteria was used to decide healthy patients? What method of sampling used to select patients?***

AR: ***Thank you for the observation. The Exclusion criteria used were as follows: < 21 years old, personal history of psychiatric illness, current use of any psychiatric medication, cardiovascular disease and any neurological disorder or brain injury in the past. We add a footnote with this information in the the section 3.1.***

RC: ***What about the ethical consideration of this study? Though it is mentioned in the Ethics Statement, it would be nice to declare in the manuscript.***

AR: ***Thank you for the observation. In the section 3.1 we add the follow paragraph: "All methodologies for this investigation were approved by the Universidade Federal de São Paulo's Ethical Committee, and the study was carried out in compliance with available criteria for human hallucinogen research safety [1]."***

[1] Johnson, Matthew W., William A. Richards, and Roland R. Griffiths. "Human hallucinogen research: guidelines for safety." Journal of psychopharmacology 22.6 (2008): 603-620.

RC: ***Figures are shown after the references, which is troublesome for the reader. I would advise authors to keep some important figures body of the writing and remaining should go to the bottom of the manuscript.***

AR: ***Thank you for the observation. We insert the most important figures in the manuscript body.***
